# Ultrasound in cardiovascular care: a perspective on preventive, diagnostic, and monitoring applications

**DOI:** 10.3389/fcvm.2026.1721700

**Published:** 2026-02-18

**Authors:** Yoram Chaiter, Daniel Lyon Fink, Yossy Machluf

**Affiliations:** 1 The Israeli Center for Emerging Technologies in Hospitals and Hospital-Based Health Technology Assessment, Shamir (Assaf Harofeh) Medical Center, Zerifin, Israel; 2 Department of Pediatric Cardiology Unit, HaEmek Medical Center, Afula, Israel; 3 Shamir Research Institute, University of Haifa, Kazerin, Israel

**Keywords:** atherosclerosis, cardiovascular ultrasound, carotid intima-media thickness, contrast-enhanced ultrasound, coronary artery disease, intravascular ultrasound, preventive cardiology, vascular imaging

## Abstract

Cardiovascular disease (CVD) remains the leading global cause of death, underscoring the need for improved strategies for early detection and prevention. Conventional risk models such as Framingham and SCORE estimate population-level risk but often fail to identify individuals with subclinical vascular damage. Atherosclerosis, the underlying cause of most CVDs, develops silently for decades, emphasizing the importance of imaging modalities capable of detecting early vascular changes. Ultrasound offers a safe, non-invasive, and radiation-free means to visualize vascular structure, function, and hemodynamics in real time. This perspective reframes ultrasound as a cornerstone of preventive and diagnostic vasculature medicine in general, and cardiovascular medicine in particular, emphasizing major modalities—point-of-care ultrasonography (POCUS), Doppler, Duplex, contrast-enhanced ultrasound (CEUS), elastography, and pulse wave velocity (PWV). These techniques enable early risk stratification, monitoring of atherosclerotic progression, and evaluation of therapeutic response across carotid, aortic, peripheral, coronary, cerebral, and renal arteries. Ultrasound also serves as a behavioral catalyst, enhancing patient awareness and engagement. However, widespread adoption requires standardized protocols, provider training, ethical oversight, and equitable implementation to avoid global disparities. Recent advances in artificial intelligence (AI), machine learning (ML), and deep learning (DL) are revolutionizing ultrasound by automating plaque quantification, improving reproducibility, and expanding access through portable and handheld systems. Cloud-based interpretation and telemedicine integration further extend cardiovascular screening into community and home settings. As ultrasound evolves into a frontline technology for prevention, diagnosis, and monitoring, its integration with AI-driven interpretation and mobile health platforms positions it as a pivotal tool in personalized and equitable cardiovascular care.

## 1 Introduction

Cardiovascular disease (CVD) remains the leading cause of death worldwide, accounting for an estimated 18 million deaths annually. Diverse CVD risk factors contribute to the progressive deterioration of the arterial vascular system, which in turn may lead to cardiovascular incidents (1–3). Conventional CVD risk scoring models, such as Framingham, SCORE, PCE, ARIC, and JHS studies, offer population-level predictions mainly focusing on a 10-year risk of CVD (4–8). However, these models lack precision at the individual level, often failing to identify patients at imminent or high risk, especially those with poor vascular health. Despite advances in therapeutic strategies, early and systematic monitoring of vascular changes—even before clinical symptoms appear—especially in vulnerable groups, may be essential for preventing CVD, facilitating early detection, effective treatment, and tailored intervention. This could potentially lead to a decrease in morbidity and mortality rates (9), but still remains an underutilized approach.

Atherosclerosis, the underlying pathology of most CVDs (10), begins silently, progresses over decades, and plays a pivotal role in the majority of deaths globally. Atherosclerosis is initiated by endothelial dysfunction, lipid accumulation, and chronic vascular inflammation (11). Emerging evidence suggests that it affects younger population, women, and a broader range of ethnic backgrounds to a greater extent than previously recognized (12). “Vascular failure” refers to a comprehensive syndrome of abnormal vascular function. It establishes atherosclerotic disease with serial events such as arterial stenosis, calcification of the vessel wall, and the consequences that plaque rupture and thromboembolic occlusion may cause (13). Physiological diagnostic criteria for several vascular functional tests, identifying possible subjects with vascular failure, were defined accordingly (14–17).

Clinical and research applications should utilize vasculature assessment modalities, which encompass key parameters related to blood vessel structure and function. Assessment of vascular structure, from anatomical perspective, refers to the arterial wall structure and thickness, as well as the calcifications and plaque characteristics. Evaluation of vascular function involves assessments of blood flow velocity, the condition and function of vascular endothelial, and blood vessels elasticity/stiffness. There is a wide range of advanced invasive and non-invasive cardiovascular assessment techniques. The latter are usually used for initial screening and for studies involving either large-scale epidemiological features or long-term follow-ups of low-risk patients. In contrast, the former are typically employed when precise data is required, or when the patient's situation is intricate or necessitates intervention. The choice of an assessment technique is driven by patient's condition and safety (susceptibility to physiological harm and psychological stress), the technique's complexity, availability, study design/management protocol, and resource requirements, etc. Therefore, non-invasive techniques are more commonly used. Among them, ultrasound stands out for its safety, accessibility, power, and versatility, solely or in combination with other modalities (18).

This perspective reframes ultrasound from a diagnostic tool to a foundational element of screening and preventive cardiovascular medicine. We emphasize clinical applications, highlight recent technological advancements, and identify systemic changes needed for widespread adoption. We also call for investment in multidisciplinary implementation and research to fully leverage its capabilities.

## 2 The subclinical landscape of atherosclerosis and ultrasound modalities

Ultrasound offers a cost-effective and real-time imaging of vascular structure and hemodynamics, allowing direct visualization of atherosclerotic changes without exposure to radiation or contrast agents (19). Its capacity to detect subclinical disease, particularly through carotid intima-media thickness (IMT) and plaque characterization, supports its role in early risk stratification. Moreover, high-frequency ultrasound enables more detailed imaging of superficial vessels, facilitating early-stage intervention. Nevertheless, it requires trained operators as poor technique, angle misalignment, poor acoustic windows, patient habitus (e.g., obesity), and calcification can degrade image or flow detection. Moreover, thresholds (e.g., velocity cutoffs) vary between devices, laboratories, stents vs. native vessels, etc., and better standardization is required. Ultrasound adoption across specialties is fragmented, and its inclusion in clinical guidelines is inconsistent.

Hereafter, we briefly list the diverse ultrasound modalities and their usage, highlighting recent advances. For complementary information on their strengths and limitations see Table 1.

**Table 1:** Key features of non-invasive ultrasound modalities.

Modality	Typical use	Type of information	Key Advantages	Key Limitations/Challenges
**POCUS** (Point-of-Care Ultrasound)	Emergency/critical care (DVT, AAA, trauma FAST), aimed at quick answer (Yes/No), rapid vascular access/interventions guidance, screening in primary/community care.	Anatomical (structure)	Portability and immediate availability, use at patient's bedside, rapid, low cost, integrating sonographic findings with medical history and clinical examination enabling real-time decision making.	Operator-dependent: need to establish formal training for general and emergency practitioners before independent use of POCUS (usually limited scans done by treating clinicians rather than radiology/sonography specialists), image quality depends on operator skills and machine type, only provides a limited field of view with lower image resolution and restricted depth penetration, often less quantitative, risk of missing subtle pathology.
**Doppler Ultrasound** (Color, Power, Spectral)	Assessing vascular stenosis (and its severity)/occlusion, venous thrombosis evaluation, hemodynamic monitoring.	Functional (flow)	Widely available and relatively inexpensive, quantifies velocity and direction, turbulence, quantifies severity of stenosis.	Limited in detecting small/deep vessels and reduced sensitivity for microvascular perfusion, flow artifacts, affected by calcification, dependency on angle and acoustic window, measurement thresholds may vary.
**Duplex Ultrasound** (B-mode + Doppler)	Carotid artery stenosis evaluation, peripheral artery disease (PAD), Venous insufficiency/mapping.	Anatomical (structure) and functional (flow)	Detailed assessment of arterial patency and blood flow, high accuracy for stenosis, non-invasive alternative to angiography.	Less optimal for calcified plaques that obscure the lumen, operator dependency and accuracy, which tend to be lower at centers with less exposure or experience, intensive and time-consuming.
**CEUS** (Contrast-Enhanced Ultrasound)	Carotid plaque neovascularization, vascular graft surveillance, detection of endoleaks.	Anatomical (structure)	Better detection of difficult-to-visualize blood flow, perfusion, and microvascular vessels, detection of intra-plaque neovascularization and perfusion abnormalities in myocardial and peripheral blood vessels, improved sensitivity and specificity, real-time dynamic assessment, higher inter-observer agreement.	Requires contrast agents, which may contraindicate in rare allergy cases, limited availability, more expensive, offers fewer choices, sometimes restricted by acoustic windows or patient habitus, regulation/approval issues in some locales, has limited standardization, and is highly operator dependent.
**Elastography** (Strain, Shear Wave)	Carotid plaque vulnerability assessment	Vascular stiffness and functional (flow)	Quantitative, reproducible, evaluates localized direct tissue stiffness measurements and direct stiffness maps offering greater precision for specific regions and lesions.	Artifacts (body movements and sound distortions, limited depth penetration—particularly in obese patients), machine/vendor variability, reliance on the operator, overlap between benign/malignant stiffness values.
**PWV** (Pulse Wave Velocity)	Cardiovascular risk stratification, monitoring vascular aging/intervention effects	Vascular stiffness	Quantifies arterial stiffness (CV risk marker), reproducible, can track treatment response, ultrafast ultrasound is effective, user-friendly.	Provides global stiffness rather than local pathology, requires special equipment, influenced by blood pressure, age and BMI, IMT and transducer type—thus all should be considered in clinical practice.

*Point-of-care ultrasonography (POCUS)*: is a diagnostic ultrasonography usually performed and interpreted at bedside by the attending physician, rather than radiology/sonography specialists (20). Recent evidence supports the use of POCUS for diagnosing abdominal aortic aneurysm and deep venous thrombosis of lower extremities (21). POCUS of carotid arteries is a highly sensitive and specific method for detecting carotid atherosclerosis, specifically among apparently healthy subjects with high and very high CVD risk (22).

*Doppler ultrasound*: uses the Doppler effect to assess the movement of blood or fluid. It gives information on flow velocity and direction. Diverse modalities—such as color Doppler or spectral Doppler, etc.—are available. Doppler velocities can support quantifying the severity of stenosis, etc (23–25). Carotid ultrasound is the primary non-invasive method for detecting, grading, and monitoring internal carotid artery (ICA) stenosis (26). Methods that were developed in the last decade add another dimension to ultrasound effectiveness in accessing microvascular flows (27).

*Duplex ultrasound*: refers to the combination of anatomic imaging and flow measurement by Doppler, which enables a detailed assessment of arterial patency and blood flow, specifically by adding functional information to anatomical imaging. Duplex ultrasonography is very useful in the follow-up of patients who underwent endovascular treatment (28–30). Recently, duplex ultrasound was used to establish normal diameter and depth values for the common femoral vasculature (31). Furthermore, renal resistive index (RRI) was established as a marker of overall cardiovascular risk in hypertensive patients, independent of renal function (32), using duplex ultrasound.

*Contrast-enhanced ultrasound (CEUS)*: uses injected micro-bubble contrast agents to enhance the contrast between vessels and background, thus improving vascular imaging sensitivity of blood vessels and plaque characteristics (33, 34). CEUS is especially valuable in evaluating difficult-to-visualize vascular territories in patients with high body mass index (BMI). CEUS has been shown to be valuable for evaluating intraplaque neovascularization (IPN) within vulnerable plaques in carotid arteries (35).

CEUS is recommended for surveillance and follow-up of abdominal aortic aneurysm endovascular repair and for imaging of suspected chronic mesenteric ischemia (36).

*Elastography*: assesses tissue stiffness, offering insights into plaque composition and vulnerability (37). Plaques that are softer and rich in lipids are more prone to rupture, whereas those that are calcified tend to be more stable (38). Of note, real-time elastographic mapping is influenced by interference from body movements and sound distortions caused by breathing, heartbeat, body fat and gas, and difficulties in measuring tissue stiffness in some patients, particularly those who are obese (due to limited depth penetration) (37). Shear wave elastography (SWE) is useful for measuring arterial stiffness and myocardial stiffness (38, 39).

*Pulse wave velocity (PWV)*: refers to the speed at which pressure waves travel along the walls of large arteries, generated by the heart's ejection of blood during each cardiac cycle. It serves as a key indicator of arterial stiffness, determined by measuring pressure or vessel diameter at two separate arterial sites (40). Of note, age, blood pressure, and BMI are all independently linked to carotid PWV. Also, IMT and transducer type affect the ability to obtain an ultrafast PWV measurement. Thus, both should be considered in clinical practice (41, 42). Recent studies found that PWV was positively associated with the risk of cardio-cerebrovascular disease (and mortality), making it a reliable and innovative predictor of these conditions (43) and a marker for arterial stiffness (44). The usefulness and accuracy of PWV in predicting cardiovascular and all-cause mortality (45), as well as in detecting coronary artery disease (CAD) (46), have already been established among high-risk populations or and among individuals with suspected disease.

While PWV is considered the gold standard for assessing overall arterial stiffness, elastography provides a complementary approach by evaluating tissue stiffness through the propagation of mechanical waves within the tissue. Unlike PWV, which reflects global arterial properties, elastography yields localized, direct stiffness measurements and detailed stiffness maps, potentially offering greater precision for specific regions such as the aorta (47, 48).

Intravascular ultrasound (IVUS) (49) enables individualized assessment of the anti-atherosclerotic therapies, and advanced tissue characterization allows direct risk stratification of coronary lesions, for example through virtual histology IVUS (50). In addition to IVUS, other intravascular modalities—such as optical coherence tomography (OCT) and near-infrared spectroscopy—enable the detection of lipid-rich plaques and demonstrated high accuracy in identifying high-risk coronary lesions (51). Nevertheless, invasive ultrasound modalities, ultrasound techniques not relevant to the cardiovascular system, and non-ultrasound imaging techniques are beyond the scope of this article.

The choice of ultrasound modality in cardiovascular care is driven by both the features of the modalities and the clinical needs. POCUS, preferably with Doppler if feasible, is best suited for screening and making quick bedside decisions. For quantifying the severity of stenosis/inflow/outflow vascular disease, one would prefer Duplex ultrasound. CEUS, used independently or combined with Doppler or Duplex, is particularly effective for monitoring grafts or stents, for detecting endoleaks or restenosis, and gaining insights into microvascular flow, and hemodynamic patterns. It is also superior for identifying plaque characteristics or assessing risk factors, such as plaque ulceration and neovascularization. For assessing vascular stiffness, PWV and elastography are the methods of choice; the former is usually used for general vascular stiffness, whereas the latter is better for assessing local vascular stiffness. In the following section, we will dive into specific cardiovascular care cases.

## 3 Clinical applications of ultrasound in cardiovascular care

### 3.1 Carotid IMT and plaque imaging

Carotid IMT/plaque is a validated surrogate marker for systemic atherosclerosis and is predictive of myocardial infarction and stroke (52). Plaque presence and morphology, including echolucency and irregular surfaces, further refine risk stratification (53, 54). Traditional ultrasound provides more detailed information than traditional risk scores alone (5, 55), though it offers only modest sensitivity.

CEUS enhances the detection of neovascularization within plaques, a marker of vulnerability (56, 57). CEUS improves delineation of lumen boundaries and highlights ulcerations and thrombi—features associated with higher risk (58). The development of real-time plaque strain imaging may also offer future risk prediction potential.

### 3.2 Abdominal aortic aneurysm (AAA) screening

Abdominal ultrasound is the gold standard for detecting AAA, particularly in asymptomatic men over aged 65–75 years, especially those with a history of smoking. Screening is also recommended for women aged 65–75 years who have ever smoked or who have a family history of AAA. Population screening programs have reduced AAA-related mortality and shown to be cost-effective (59, 60).

Ultrasound is also central to post-endovascular aneurysm repair (EVAR) surveillance, where it is used to detect endoleaks, assess graft integrity, and monitor aneurysm size (61). Advanced Doppler techniques allow for improved hemodynamic assessment in aneurysm surveillance.

### 3.3 Peripheral arterial disease (PAD) evaluation

Duplex ultrasound enables a detailed assessment of arterial patency and blood flow in the lower extremities. It identifies the site and severity of the obstruction and it provides information on revascularization strategies (62). Its role extends to follow-up after interventions, where it can detect restenosis or graft failure. While systemic mapping of peripheral arteries may be time-consuming, miniaturized portable duplex probes may offer a solution.

### 3.4 Coronary and cerebral vasculature

Although limited in imaging distal coronary arteries, recent developments in ultrafast ultrasound and CEUS have enhanced visualization of proximal segments and perfusion dynamics (63, 64). Transthoracic echocardiography (TTE), when optimized, can visualize all three major coronary arteries in selected patients (65–67).

Transcranial Doppler (TCD) remains a useful tool in evaluating cerebral hemodynamics, emboli detection, and intracranial stenosis. However, its utility is limited by poor acoustic windows and operator dependency (68–70). Advances in contrast agents and multiplanar insonation may enhance TCD's diagnostic value.

### 3.5 Renal arteries

The duplex ultrasound is commonly used to assess the renal blood vessels and is successful in identifying renal artery stenosis (RAS) (71). CEUS has also displayed high diagnostic accuracy for pinpointing RAS when compared with angiography (72).

Ultrasound's ability to visualize various vascular beds varies based on anatomical depth (including skull thickness), acoustic window availability, technique, and sometimes also patient demographics. Understanding these anatomical and technical nuances is essential for optimizing ultrasound preventive utility in identifying subclinical vascular disease across diverse patient populations (Table 2).

**Table 2:** Major arteries visualization success rates by ultrasound.

Arteries	Methods	Success rates (SR)	References
Middle Cerebral Arteries (MCA)	TCD	SRs ranging 65%–81%, with sensitivity >90% and specificity of 100% for MCA stenosis	(69, 70, 124)
Coronary Arteries	TTE	SR of proximal left main coronary artery (LMCA): 86%; left anterior descending artery (LADA): 86%; right coronary artery (RCA): 90%, while circumflex artery remains more challenging, demonstrating only 30%. Pediatric visualization rates reach ∼95%.	(63, 65)
AAA & Iliac Arteries	Ultrasound	AAA detection sensitivity exceeds 95%. Robotic ultrasound platforms demonstrate 100% accuracy in pilot studies.	(59, 60)
Renal Arteries	Duplex ultrasound	Proximal renal artery stenosis (RAS): ∼90%–95% sensitivity and 85%–90% specificity, with full artery course visualization in 65%–76%.	(125–128)
	CEUS	High overall diagnostic accuracy, sensitivity and specificity to detect RAS were noted (comparable to the corresponding values of angiography).	(72)
Mesenteric & Celiac Arteries	Duplex ultrasound	Sensitivity, specificity and overall accuracy for superior mesenteric artery (SMA), celiac artery (CA) and inferior mesenteric artery (IMA) stenosis (>50%) were 90%/91%/91%, 93%/100%/95% and 90%/96%/95%, respectively.	(129, 130)
Peripheral Lower Limb Arteries	Duplex ultrasound	Sensitivity and specificity for significant stenosis (>50%) are ≥85%–90%. Sensitivity and specificity of Duplex ultrasound for the diagnosis of arterial lesions in the entire lower limb 88% (80–98%) and 96% (89–99%)	(28, 131)

## 4 Monitoring atherosclerosis progression and therapeutic response

The non-invasive and repeatable nature of ultrasound makes it ideal for serial assessments. Changes in IMT or plaque size can reflect the effects of lipid-lowering, antihypertensive, or antidiabetic therapies (73). Ultrasound is used to track vessel patency, flow dynamics, and potential complications in post-revascularization care.

Its utility extends to high-risk populations, such as patients with familial hypercholesterolemia or diabetes, where close monitoring may influence therapeutic decisions. Moreover, integration with electronic health records enables longitudinal tracking of vascular changes, improving real-world clinical utility.

## 5 Advances in vascular ultrasound

Artificial Intelligence (AI) has been increasingly employed to revolutionize prevention, diagnosis, treatment, prognosis predication and risk assessment, clinical care, and drug discovery in the field of cardiovascular medicine (74, 75).

Combining advanced algorithmic approaches like AI, machine learning (ML), deep learning (DL), automation, and pattern recognition into sonographic analysis allows for new vascular imaging opportunities—from image acquisition, automation, segmentation and interpretation, through diagnostics, therapy planning, and prognostication, and to integration with other clinical data while enhancing accessibility, accuracy, speeding up analysis and reporting, and reducing the workload (76–82). ML, a subset of AI, employs algorithms capable of identifying patterns within training data and applying them to generate accurate predictions for new data. DL, an advanced branch of ML, leverages multi-layered neural networks to process complex inputs, such as medical images, text, and audio, thereby improving the precision, range and scope of image interpretation (83). AI-based systems for cardiovascular imaging might be viewed as a tool to augment clinical decision-making and improve workflow efficiency (79). Yet, despite the promises of AI application to cardiovascular imaging, some challenges remain (79–82, 84).

### 5.1 Ultrasound modalities, AI and CVD diagnosis

AI has numerous applications in echocardiography, including guided image acquisition for optimal imaging, automated quantification of cardiac function, and disease detection and classification. It can also enhance strain analysis and 3D echocardiography, improve risk stratification, and optimize clinical workflow, potentially leading to faster, more accurate assessments, and streamlined decision-making. Moreover, AI algorithms can cross-reference imaging results from echocardiography, cardiac computed tomography (CCT), or cardiovascular magnetic resonance (CMR) with a patient's clinical records, lab results, and genetic information thus organizing vast amounts of information into a more interpretable format and aiding physicians to reach correct diagnosis (82, 85–87).

AI-enabled systems can reduce operator dependency, improve image quality, and promote consistency across diverse clinical environments. Challenges include algorithm generalizability, bias, explainability, clinician trust, and data privacy. Standardized development, ethical oversight, and clinician-AI collaboration are crucial for effective implementation. Emerging innovations-such as autonomous scanning, real-time predictive analytics, tele-ultrasound, and patient-performed imaging-underscore the transformative potential of AI-enabled POCUS in reshaping cardiovascular care and advancing equitable healthcare delivery worldwide (88).

The feasibility, applicability and accuracy of AI in the detection of carotid artery disease in greyscale static duplex ultrasound images have been demonstrated (89).

CEUS is indispensable in AAA screening and post-endovascular aortic aneurysm repair surveillance, especially in patients with renal impairment. Emerging technologies, including hybrid imaging, radiomics, and AI enhance detection of subtle imaging features, automate measurements, and may enable prediction of disease progression or complications (90).

AI-based models, with elastography, have shown strong risk predictive capabilities of Metabolic Dysfunction-Associated Steatotic Liver Disease (MASLD)—a predictor of metabolic changes associated with atherosclerosis risk as well as future liver conditions such as cirrhosis (91).

Real-time assessment of vascular stiffness, pulse wave patterns, enable early cerebrovascular compromise-detection previously inaccessible with traditional, intermittent evaluation methods. By integrating AI with enhanced, continuous data acquisition from the carotid artery, new diagnostic and predictive pathways enable, precision-based care and improved patient outcomes such as stroke prevention, by real-time cerebrovascular monitoring, and broader vascular assessment (92).

### 5.2 AI driven interpretation and 3D imaging

AI algorithms reduce operator variability and enhance diagnostic consistency by automating plaque measurements and flow analysis (93, 94). Three-dimensional ultrasound facilitates volumetric plaque assessment, improving reproducibility and clinical confidence. Cloud-based AI platforms may soon enable remote expert consultation (95).

### 5.3 AI, ML and DL algorithms for quantitative imaging

Automated segmentation algorithms facilitate consistent measurement of cardiac chambers, ejection fraction, and wall motion abnormalities while minimizing operator dependence (96, 97). These algorithms also enable reproducible quantification of vascular structures, which supports the widespread adoption of carotid IMT as a biomarker for atherosclerosis. Automated IMT measurement decreases inter-observer variability and enhances its utility in large-scale screening for risk stratification (98, 99).

ML models can also support predictive analytics by integrating ultrasound findings with clinical data to refine cardiovascular risk assessment. Quantitative ultrasound phenotyping, such as speckle-tracking strain analysis, combined with AI-based interpretation, has demonstrated potential for the early detection of subclinical myocardial dysfunction and vascular stiffening (100, 101).

DL in CVD diagnosis, treatment planning, and prognostic modeling, can reduce unnecessary diagnostic imaging, predict high-cost complications, and optimize the utilization of critical resources like intensive care unit (ICU) beds (102).

### 5.4 Automated plaque characterization

Plaque morphology, rather than luminal stenosis alone, is now recognized as a critical cardiovascular risk determinant. While conventional ultrasound offers grayscale and Doppler data, AI-enhanced plaque characterization enables the automated classification of plaques as stable or vulnerable by analyzing echogenicity, surface irregularity, and neovascularization patterns. ML algorithms trained on extensive image datasets have demonstrated high accuracy in detecting lipid-rich or ulcerated plaques, which are predictive of ischemic events (103, 104). These technological advances may facilitate scalable, non-invasive monitoring of atherosclerotic disease progression for primary prevention. Furthermore, use of AI technology for the segmentation of plaques in ultrasound images, and analysis of radiomics models, to determine stability of carotid artery plaques, provides a diagnostic basis for the clinical prediction of ischemic stroke (105). AI-based methods for IMT and plaque area segmentation—including detection and measurement from ultrasound images by ML and DL—have emerged for CVD/stroke risk monitoring and for atherosclerosis assessment, of both the carotid (106, 107) and coronary arteries (108).

### 5.5 Pattern recognition in CEUS and Doppler imaging

Currently, AI-driven pattern recognition is applied to CEUS time–intensity curves and Doppler spectral signatures, enabling the automated detection of abnormal perfusion or turbulent flow patterns (109, 110). Preliminary studies have indicated that these tools can assist in identifying unstable plaques and optimizing triage for invasive angiography or intervention. These methods are particularly promising for community screening programs with limited access to specialist expertise.

### 5.6 Portable and handheld devices supported by AI tools

Nowadays, portable and handheld ultrasound devices are examples of cost-effective mobile health technology in the hands of physicians, alongside digital stethoscopes, etc. (111). These devices provide diagnostic accuracy comparable to conventional systems in assessing left ventricular dysfunction, valvular disease, basic vascular imaging, and in detecting several pathologies (112–114). These systems also support treatment decisions based on feedback from image acquisition and interpretation processes (115), as they enable asynchronous consultation, with images uploaded for review by specialists, reducing inequities in access to cardiovascular imaging (116, 117). However, they are limited by a narrow field of view, inadequate penetration, and poor visualization of solid organ parenchymal disease.

The incorporation of AI, encompassing DL and ML, into portable and handheld compact ultrasound systems—either through on-device integration or cloud-based interpretation via smartphone connectivity—represents a significant advancement. This approach enhances diagnostic capabilities, facilitates remote monitoring, supports teleconsultation, and enables non-specialists to perform reliable community-based screenings, namely real-time image acquisition, transmission, and cloud-based interpretation (93, 114), thereby expanding the clinical impact of ultrasound technologies, particularly in underserved or geographically remote settings. These devices coupled with AI guidance are critical for democratizing access to vascular health technologies.

## 6 Ultrasound in preventive cardiology and risk reclassification

Ultrasound refines cardiovascular risk estimation by identifying patients with subclinical atherosclerosis not captured by conventional models (68, 118). Studies have shown that carotid plaque detection can reclassify 10%–20% of patients at intermediate risk to a higher risk category, altering management strategies (119).

As demonstrated in pregnancy care, the potential for democratizing ultrasound self-screening in the population is now feasible (120). Screening for endovascular pathology and AAA can be performed by local village health care workers in third world countries, or as a neighborhood project in any country, thanks to combined small and low-cost ultrasound transducers and government-supported software that employs AI guidance, running on personal electronic devices, to replicate the correct views and automated results (88).

Incorporating ultrasound metrics into existing algorithms could enhance risk discrimination and individualize preventive therapy, aligning with personalized medicine initiatives. Integration of vascular ultrasound findings into digital health platforms for shared decision-making should be explored in future efforts.

## 7 Ultrasound as a behavioral catalyst

Clinical guidelines have increasingly recognized the role of vascular ultrasound, recommending its use for risk assessment, screening, and monitoring in selected populations (121). Recent literature advocates for even more comprehensive vascular evaluations and multidisciplinary approaches, positioning ultrasound as a cornerstone of modern vascular medicine (9). We propose the integration of ultrasound imaging, using standardized protocols, via cross-disciplinary collaborations, into care pathways of patient counseling sessions to enhance awareness and engagement in disease prevention. Clinicians should receive communication training to effectively convey ultrasound findings and their implications. Recently, an imaging-based cardiovascular risk-reduction program (WAKE UP) was established to raise awareness of women's CVD and promoting lifestyle changes (122). In this longitudinal, prospective, case-control study, vascular ultrasound imaging—including 2D assessment of the carotid, aorto-iliac, and femoral arteries and 3D evaluation of any detected plaques—can help identify subclinical atherosclerosis in a visible and actionable way, potentially motivating greater adherence to preventive strategies among women with plaques.

### 7.1 Implementation and equity considerations

Despite these technological advances, deployment remains uneven. Handheld and AI-supported ultrasound is uniquely positioned to extend diagnostic capability into underserved communities with high CVD burden. Pilot programs have demonstrated the feasibility of community-based vascular and cardiac screening using mobile ultrasound units or task-shared protocols (121, 123).

However, widespread adoption requires not only technical validation but also sustainable integration into healthcare systems and robust quality assurance. Training non-specialist providers in POCUS is a growing trend, with family physicians, emergency doctors, and paramedics increasingly performing basic cardiovascular assessments under remote supervision (9).

Ensuring equitable deployment is challenging—while handheld devices are inexpensive relative to traditional systems, sustainable financing and infrastructure support are required to avoid widening global disparities.

### 7.2 Ethical and regulatory challenges

The rapid expansion of cardiovascular ultrasound raises important ethical and regulatory considerations. Overdiagnosis and misdiagnosis are both potential harms, particularly when AI tools are applied without sufficient clinical oversight. False positives may generate unnecessary anxiety and downstream testing, whereas false negatives could delay care. Regulatory frameworks for handheld devices and AI-based interpretation remain fragmented, with insufficient guidance available on validation, liability, and their integration into clinical practice (34, 64).

## 8 Future directions and concluding remarks

Ultrasound has evolved from being a supplementary diagnostic tool to a versatile modality in the prevention, diagnosis, and longitudinal monitoring of CVD. Its combination of safety, accessibility, and adaptability positions it as a pivotal technology in addressing the global burden of atherosclerosis.

Cardiovascular ultrasound is undergoing a profound transformation fueled by technological innovation, device miniaturization, and the integration of AI. Once confined to specialized hospital settings, ultrasound is now moving closer to the patient — into point-of-care environments, community clinics, and even homes. This shift carries major implications for screening, diagnosis, monitoring, and the delivery of personalized cardiovascular care.

To fully realize its potential, further work is needed to validate AI-driven interpretation platforms, automate IMT and plaque quantification, evaluate cost-effectiveness across diverse health systems, and assess the long-term impact of ultrasound-based counseling. The feasibility of home-based or self-administered vascular ultrasound also warrants exploration, particularly in conjunction with wearable biosensors for real-time cardiovascular monitoring.

As cardiovascular ultrasound evolves into a frontline tool, balancing technological progress with responsible deployment will shape its ultimate role in global cardiovascular prevention and care. Ethical oversight, regulatory clarity, and equitable implementation are critical to ensuring that innovation translates into meaningful health benefits. With coordinated efforts in research, guideline integration, training, and innovation, ultrasound has the potential to become a true cornerstone of personalized cardiovascular medicine.

## Data Availability

The original contributions presented in the study are included in the article/supplementary material, further inquiries can be directed to the corresponding author.
